# Serum Procalcitonin and Procalcitonin Clearance as a Prognostic Biomarker in Patients with Severe Sepsis and Septic Shock

**DOI:** 10.1155/2016/1758501

**Published:** 2016-03-20

**Authors:** Min-Yi Huang, Chun-Yu Chen, Ju-Huei Chien, Kun-Hsi Wu, Yu-Jun Chang, Kang-Hsi Wu, Han-Ping Wu

**Affiliations:** ^1^Department of Medicine, Taichung Tzu Chi Hospital, the Buddhist Medical Foundation, Taichung 42743, Taiwan; ^2^Division of Emergency Medicine, Department of Pediatrics, Changhua Christian Hospital, Changhua 500, Taiwan; ^3^School of Medicine, Chung Shan Medical University, Taichung 402, Taiwan; ^4^School of Medicine, Kaohsiung Medical University, Kaohsiung 807, Taiwan; ^5^Department of Laboratory Medicine, Taichung Tzu Chi Hospital, the Buddhist Medical Foundation, Taichung 42743, Taiwan; ^6^Laboratory of Epidemiology and Biostatistics, Changhua Christian Hospital, Changhua 500, Taiwan; ^7^School of Post-Baccalaureate Chinese Medicine, College of Chinese Medicine, China Medical University, Taichung 404, Taiwan; ^8^Department of Hematology-Oncology, Children's Hospital, China Medical University Hospital, China Medical University, Taichung 404, Taiwan; ^9^Division of Pediatric General Medicine, Department of Pediatrics, Chang Gung Memorial Hospital at Linkou, Kweishan, Taoyuan 333, Taiwan; ^10^College of Medicine, Chang Gung University, Taoyuan 333, Taiwan

## Abstract

We evaluated the tendency of the plasma concentration and procalcitonin (PCT) clearance (PCTc) to act as biomarkers of prognosis in patients with severe sepsis and septic shock. From 2011 to 2013, we prospectively analyzed patients with sepsis admitted to the intensive care unit (ICU). The serum PCT was evaluated at the time of sepsis diagnosis and again after 48 h (day 3) and 96 h (day 5). PCTc after 48 h (PCTc-day 3) and 96 h (PCTc-day 5) was also calculated to evaluate the prognostic value for survival in patients with sepsis. A total of 48 patients were included. Overall mortality was 16.7% (8 patients). PCTc was higher in survivors than in nonsurvivors, with significant differences on day 3 and day 5 (*p* = 0.033; *p* = 0.002, resp.); however, serum PCT levels on day 1, day 3, and day 5 were not significant prognostic factors for survival. The prognosis of patients with severe sepsis and septic shock may be associated with PCTc. Dynamic changes of PCT reflected as PCTc at 48 h (day 3) and 96 h (day 5) after admission to the ICU may serve as a predictor of survival in critically ill patients with severe sepsis.

## 1. Introduction

Sepsis is one of the leading causes of mortality in intensive care units (ICUs) [1]. The early detection of patients with sepsis in the ICU with worsening prognosis or with an increased risk of mortality is essential to prevent consequent organ dysfunction. Prompt diagnosis and administration of appropriate antimicrobial therapy are essential to reduce complications associated with sepsis-related organ failure and patient mortality; however, the individual response to sepsis is complex and not all patients with infections show signs or symptoms [2, 3]. In this context, it is useful to have biomarkers that can serve as predictors for sepsis in clinical practice.

Procalcitonin (PCT) is the 116-amino acid long precursor of calcitonin, which is elevated in sepsis [4, 5]. The degree of induction of PCT is associated with the severity of systemic infection and the presence of organ dysfunction. Thus, PCT is regarded as a useful biomarker for the diagnosis of sepsis, and recent studies have suggested that dynamic changes of PCT could be predictive of certain outcomes in patients with severe sepsis and septic shock. Serum PCT can aid in the diagnosis of sepsis in critically ill patients; however, its prediction for survival is not well established, and a dynamic approach of assessing biomarkers may provide additional survival information of septic patients [4, 6]. A concept of PCT clearance (PCTc) has been introduced in a pilot study as a tool for monitoring the evolution of PCT levels during severe sepsis [4–6]. PCTc measures the relative changes in PCT to the baseline PCT and is postulated to be a better predictor of outcome. Therefore, the hypothesis of this study is whether PCT levels and PCT clearance could serve as prognostic biomarkers for patients with severe sepsis and septic shock. The aim of the present study was to evaluate the usefulness of PCT levels and PCTc as biomarkers of prognosis in patients with severe sepsis and septic shock.

## 2. Materials and Methods 

### 2.1. Patient Population

This is a prospective observational study conducted in the medical ICUs at a hospital in central Taiwan from July 2011 to June 2013. In the present study, all consecutive patients who fulfilled the criteria for sepsis were enrolled. Patients who underwent surgery or trauma or were aged less than 18 years were excluded from our series. The study was approved by the Institutional Review Board of the Taichung Tzu Chi Hospital, and a written informed consent was obtained from patients or patient representatives. Patients were treated according to the institutional protocol for the management of severe sepsis and septic shock, based on recommendations from the surviving sepsis campaign modified to meet recent evidence from the literature [7–9].

### 2.2. Study Design

This was a single-center, prospective observational study. All enrolled patients with sepsis or septic shock were managed based on the decision of the treating physicians in the ICU. In addition, the duration of antimicrobial therapy was guided by culture data, site of infection, and treating physician. The patients data included age, gender, vital signs, clinical status, Acute Physiology and Chronic Health Evaluation (APACHE II) score, Sequential Organ Failure Assessment (SOFA) score, site(s) of infection, laboratory tests findings (basic biochemistry, complete blood count, coagulation, and arterial blood gases), microbiological culture results, duration of hospitalization (length of stay in ICU and hospital), and clinical outcomes. Infection was diagnosed by standard clinical, laboratory, and microbiological parameters. APACHE II scores and SOFA scores on the first day after admission (day 1) and serum PCT levels on days 1, 3, and 5 of diagnosis of sepsis were calculated. The patients were followed up until hospital day 28, and the outcome at day 28 was noted as the primary outcome, defined as mortality related to sepsis within the first 28 days of admission to the ICU.

### 2.3. Measurement of Biomarkers

PCT was measured in serum samples that were collected on hospital days 1, 3, 5, and 7 in patients with sepsis and septic shock. The blood was drawn and centrifuged at 3,500 rpm for 5 min and biochemistry markers were analyzed immediately. The serum samples were then stored at −80°C until further analysis. The concentration of PCT was measured by using a one-step immunoassay sandwich method with a fluorescent detection (ELFA). The PCT detection range is 0.05–200 ng/mL according to the manufacturer's instructions (VIDAS II, Biomerieux Inc., France) [5–8]. Moreover, PCTc is calculated using the following formula [4]:

(1) PCTday  3/day  5−PCTday  1PCTday  1×100%=PCTcday  3/day  5%.

PCTc on day 3 (PCTc-day 3) and that on day 5 (PCTc-day 5) was calculated based on this formula.

### 2.4. Statistical Analysis

Data of categorical variables were analyzed by the chi-square test or Fisher's exact test, when appropriate. Continuous variables were analyzed by the Mann-Whitney *U* Test and Kruskal-Wallis Test. A *p* value less than 0.05 was considered to be statistically significant. Distributions of variables were reported as percentages and means ± standard deviation (SD). Statistical analyses were performed with SPSS software (version 15.0, SPSS, Inc., Chicago, IL, USA).

## 3. Results

### 3.1. Demographics and Clinical Presentations

During the study period, a total of 56 patients with sepsis and septic shock were admitted to the ICU at our hospital. Eight patients were excluded from the study because of incomplete clinical data, and 48 patients with sepsis were, thus, enrolled in our study. The mean age was 74 ± 12 years with 47.9% male and 52.1% female patients. On day 1, the mean APACHE II score was 22.9 ± 6.9 and the mean SOFA score was 7.2 ± 3.3. The positive blood culture rate was 60.4%. The average length of ICU stay was 12.5 ± 9.1 days, and the average total length of hospital stay was 23.0 ± 14.9 days. Ten patients required endotracheal tube insertion (20.8%), and the overall mortality was 16.7% (8 patients, 7 of whom had septic shock).

**Figure 1:**
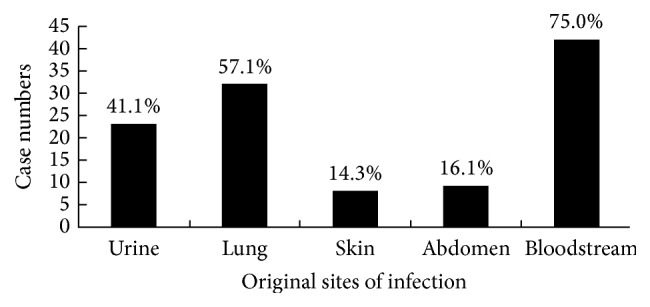
Original sites of infection identified in patients with sepsis. The most common site of infection was bloodstream infection (75.0%), followed by lung (57.1%), urine (41.1%), abdomen (16.1%), and skin (14.3%).

The original sites of infection are shown in Figure 1. Blood was identified as the most common site of infection in patients with sepsis in the ICU. The majority of sepsis (75%) was because of bacteremia. The other original sites of infection were the lungs, urine, abdomen, and skin (57.1%, 41.1%, 16.1%, and 14.3%, resp.). The isolation rate of Gram-negative bacteria was 69%.* Escherichia coli* (*E. coli*) and* Klebsiella pneumoniae* (*K. pneumoniae*) were identified as the two most common microorganisms. Gram-positive bacteria and fungus composed 18.4% and 12.6%, respectively; coagulase negative* Staphylococci* were identified as the most common Gram-positive bacteria, and* Candida albicans* was the most common fungi isolated from sputum culture. A more detailed description of microorganisms isolated from different sites in patients with sepsis is presented in Table 1.

### 3.2. Clinical Factors Associated with Survival

The results of clinical factors between survivors and nonsurvivors of patients with sepsis or septic shock are listed in Table 2. The significant factors associated with survival in patients with sepsis included APACHE II scores, positive or negative sputum culture, and the length of ICU stay. Regarding the severity of illness, APACHE II and SOFA scores were compared in the two groups. Both scores were higher in the nonsurvivors, but only APACHE II scores showed a significant difference (*p* = 0.019). Among all the results of culture data, the presence of a positive sputum culture was significantly predominant in the nonsurvivor group (*p* = 0.01). In addition, we found that survivors tended to have shorter ICU stays but longer overall hospital stays than nonsurvivors. A shorter duration of ICU stay was also a significant factor associated with survival in patients with sepsis in the ICU (*p* = 0.040).

In patients with sepsis, however, serum PCT levels on days 1, 3, and 5 were not significant prognostic factors for survival. PCT concentration showed decay from baseline in the survivors, whereas it remained high in those who did not survive. Comparison between the changes in PCT levels in survivors and nonsurvivors is shown in Table 3. PCTc was also compared between the two groups. PCTc-day 3 and PCTc-day 5 were both significantly higher in patients who survived than in those who did not (*p* = 0.033, *p* = 0.002, resp.). The PCTc on day 3 and day 5 was associated with the prediction of survival in patients with sepsis in the ICU.

## 4. Discussion

Severe sepsis with septic shock is a major cause of morbidity and mortality in the ICU. Mortality increases with the severity of sepsis. In-hospital mortality rates for severe sepsis and septic shock are high, ranging between 18% and 50% [10–12]. In the present study, the mortality because of severe sepsis and septic shock was 22.9%, which is consistent with prior results [10–12]. Clinically, once the diagnosis of sepsis is made, the prediction of survival is important for the risk stratification of patients and in indicating the potential success or failure of treatment. We evaluated the predictive value of serum PCT for survival in patients with severe sepsis or septic shock. In this prospective study of patients with sepsis, we demonstrated that serum PCT measured on days 1, 3, and 5 of ICU stay was not predictive of mortality. Measurement of biomarkers at a single time point may be of limited value because of the large variability of biomarker secretion at different times during the progression of critical illness. Moreover, it is unclear how much time has lapsed between the initial onset of disease and the time of admission to the ICU. Nevertheless, in our study, PCTc increased progressively in surviving patients but decreased in nonsurvivors, with significant differences at 48 h and 96 h (PCTc-day 3 and PCTc-day 5). In patients with severe sepsis and septic shock, dynamic changes in PCTc-day 3 and -day 5 predicted survival. There was higher survival in septic patients with increased PCT clearance on day 3 of more than 38% compared to those below 12%. In addition, patients with increased PCT clearance on day 5 of more than 80% compared to those below 44% may have higher survival rate. As we know, a dynamic approach to biomarkers may capture the progression of disease and may be more useful in evaluating patients with sepsis. In this context, we observed that serum PCT levels measured at different time points after admission were not predictive of mortality; however, dynamic changes of PCT over 48 h and 96 h were predictive. The predictive significance for using PCT in prognosis may be apparent after determination of the progression of serial PCT concentrations relative to the baseline.

In our study, the most common original site of infection in patients with sepsis was lung, followed by urinary tract and intra-abdominal infection. The majority of isolated microorganisms were Gram-negative bacteria, in which* E. coli* and* K. pneumoniae* were the most commonly identified strains. Among the isolated Gram-positive bacteria,coagulase negative* Staphylococci* were the most commonly identified strains. In our study, positive blood cultures were more common in dead patients than survivors (75% versus 57.5%), but the *p* value did not show significance. We think the results without statistically significant difference may be due to the small sample size in our study. We believe that the difference of positive blood cultures between survivors and dead patients may be very important, but, in previous studies, culture positivity could not serve as a predictor of mortality in patients with sepsis [13–15]. In our study, the presence of a positive sputum culture was significantly more predominant in the nonsurvivors group. Thus, the positive sputum culture may appear to be a predictor of prognosis, whereas other culture results may or may not be associated with mortality in patients with sepsis.

Several prognostic indices are used in ICUs. The two most widely used are the APACHE II score and SOFA score; the utility of those is limited to the first 24 h of treatment in other studies [16, 17]. In our study, we found that while both APACHE II and SOFA scores were higher in the nonsurvivors than those in survivors, only APACHE II scores showed a significant difference between the two groups. The APACHE II score was a predictor of mortality in our analysis.

In conclusion, the prognosis of patients with severe sepsis and septic shock may be associated with PCTc. Dynamic changes of PCT reflected as PCTc at 48 h (day 3) and 96 h (day 5) after admission to the ICU may serve as a predictor of survival in critically ill patients with severe sepsis. This could assist primary care physicians in the risk stratification of critically ill patients with severe sepsis and septic shock.

## Figures and Tables

**Table 1:** Microorganisms isolated from different sites in patients with sepsis or septic shock.

Category (total positive isolations *n* = 87)	Blood	Urine	Sputum	Skin	Abdomen
Gram-positive bacteria (*n* = 16, 18.4%)					
*Staphylococcus aureus*	0	0	1	1	0
Coagulase negative* Staphylococci*	8	0	0	0	0
*Streptococcus *spp.	2	0	0	0	0
*Enterococcus *spp.	1	2	0	1	0
Gram-negative bacteria (*n* = 60, 69.0%)					
*Escherichia coli*	7	10	2	2	1
*Klebsiella pneumoniae*	8	4	5	0	1
*Proteus mirabilis*	1	1	1	1	0
*Salmonella D1*	1	0	0	0	0
*Enterobacter *spp.	2	1	1	0	0
*Haemophilus influenzae*	0	0	3	0	0
*Pseudomonas aeruginosa*	1	1	2	1	0
*Acinetobacter baumannii*	0	0	1	0	0
Other *Anarobes* spp.	2	0	0	0	0
Fungus (*n* = 11, 12.6%)					
*Candida albicans*	1	2	8	0	0

**Table 2:** Comparisons of baseline characteristics between survivors and nonsurvivors in patients with sepsis or septic shock.

Variables	All patients (*n* = 48)		*p *value
	Survivors (*n* = 40)	Dead (*n* = 8)	
Gender			0.897
Male	19 (47.5)	4 (50.0)	
Female	21 (52.5)	4 (50.0)	
Culture results			
Blood culture			0.356
Positive	23 (57.5)	6 (75.0)	
Negative	17 (42.5)	2 (25.0)	
Urine culture			0.895
Positive	16 (40.0)	3 (37.5)	
Negative	24 (60.0)	5 (62.5)	
Sputum culture			0.010
Positive	15 (37.5)	7 (87.5)	
Negative	25 (62.5)	1 (12.5)	
Endotracheal tube	7 (17.5)	3 (37.5)	0.204
	Mean ± SD	Mean ± SD	
Age (years)	75 ± 11	70 ± 14	0.355
Clinical scoring			
APACHE II score (day 1)	21.9 ± 6.8	28.1 ± 5.2	0.019
SOFA score (day 1)	6.8 ± 3.2	9.0 ± 3.2	0.089
Laboratory tests			
Serum PCT (mg/dL)			
Day 1	41.4 ± 54.7	37.4 ± 66.9	0.856
Day 3	28.5 ± 44.6	33.6 ± 48.0	0.773
Day 5	11.1 ± 22.6	11.8 ± 11.1	0.925
Blood sugar (mg/dL)	218.6 ± 153.9	241.7 ± 292.5	0.745
Blood pH	7.3 ± 0.1	7.3 ± 0.1	0.299
Blood bicarbonate	18.8 ± 5.8	19.0 ± 4.9	0.932
Duration of hospitalization			
ICU stay (days)	11.2 ± 8.6	18.5 ± 9.6	0.040
Hospital stay (days)	23.9 ± 15.6	18.5 ± 9.7	0.352

**Table 3:** PCTc between survivors and nonsurvivors in patients with sepsis.

Variables	Survivors (*n* = 37)		Dead (*n* = 11)		*p* value
	Value	95% CI	Value	95% CI	
PCTc-day 3	38.9	(−154.1 to 95.1)	11.6	(−533.6 to 34.0)	0.033
PCTc-day 5	80.3	(3.5 to 99.3)	43.2	(−85.2 to 83.4)	0.002

PCTc-day 3: PCT clearance at 48 h after admission (%); PCTc-day 5: PCT clearance at 96 h after admission (%).
